# Diverse effects of pan-ROCK and ROCK2 inhibitors on 2 D and 3D cultured human trabecular meshwork (HTM) cells treated with TGFβ2

**DOI:** 10.1038/s41598-021-94791-4

**Published:** 2021-07-27

**Authors:** Megumi Watanabe, Yosuke Ida, Hiroshi Ohguro, Chiaki Ota, Fumihito Hikage

**Affiliations:** Department of Ophthalmology, Sapporo Medical University School of Medicine, Sapporo, Japan

**Keywords:** Cell biology, Ocular hypertension, Optic nerve diseases

## Abstract

A pan-ROCK-inhibitor, ripasudil (Rip), and a ROCK2 inhibitor, KD025, were used To study the effects of Rho-associated coiled-coil containing protein kinase (ROCK)1 and 2 on two-dimensional (2D) and three-dimensional (3D) cultures of a TGFβ2-treated human trabecular meshwork (HTM) cells. In the presence of 5 ng/mL TGFβ2, the effects of these inhibitors were characterized by transendothelial electrical resistance (TEER), FITC-dextran permeability, and the size and stiffness of 3D sphenoids, the expression of extracellular matrix (ECM) including collagen1, 4 and 6, and fibronectin, α-smooth muscle actin, a tissue inhibitor of metalloproteinase (TIMP)1–4, and matrix metalloproteinase (MMP)2, 9 and 14. TGFβ2 caused a significant increase in the TEER values, and decrease in FITC-dextran permeability, as well as a decrease in the sizes and stiffness of the 3D sphenoids. In the presence of ROCK inhibitors, the TGFβ2-induced effects of the TEER and FITC-dextran permeability were inhibited, especially by KD025. Rip induced a significant increase in sizes and a decrease in the stiffness of the TGFβ2-treated 3D sphenoids, although the effects of KD025 were weaker. Gene expressions of most of the ECMs, TIMP2 and MMP9 of 2D and 3D HTM cells were significantly up-regulated by TGFβ2. Those were significantly and differently modulated by Rip or KD025.

## Introduction

Rho-associated coiled-coil containing protein kinases (ROCKs) among the serine-threonine protein kinase family are well recognized as regulators of the remodeling of the actin cytoskeleton^1–5^. Two types of ROCKs, ROCK1 (ROKβ) and ROCK2 (ROKα) are composed of homologous amino acid compositions of the carboxyl termini, the catalytic kinase domain and the Rho-binding domain (RBD) in addition to a distinct coiled-coil region^6,7^. ROCK1 and ROCK2 are functionally involved in the regulation of the organization of the actin cytoskeleton, differentiation, apoptosis, glucose metabolism, cell adhesion/motility, and inflammation^8–10^. Within ocular and peri-ocular tissues, ROCKs are also expressed, including the trabecular meshwork, ciliary muscles, and the retina^6,7^, and play pivotal roles in the ocular pathophysiology in several ocular diseases such as cataracts, retinopathy, and corneal dysfunction^1,2,11–14^. Based upon these observations, ROCKs are paid great attention to become therapeutic targets for these ocular diseases. In fact, several studies revealed that ROCK inhibitors (ROCK-is) have hypotensive effects toward intraocular pressure (IOP) in several animal models^15,16^, and one of the ROCK-is, ripasudil hydrochloride hydrate (Rip), a non-selective ROCK-i, is already available as an anti-glaucoma medications for the treatment of glaucoma and ocular hypertension^17,18^. We recently developed three-dimension (3D) drop cell cultures using human TM (HTM) in conjunction of TGFβ2, which was revealed to increase transcellular pressure and suppress ease of outflow^19^, to replicate a relevant ex vivo glaucoma TM model^20^. Using this model, we found that TGFβ2 induced significantly smaller and stiffer 3D HTM sphenoids, and such TGFβ2 induced effects were substantially reduced by the presence of the pan ROCK inhibitors, Rip or Y27632^20^. However, conjunctival hyperemia and others are recognized as unknown caused as adverse periocular side effects in the Rip and other ROCK-is^17,18^. Since, as described above, ROCKs, ROCK1 and ROCK2, differently contribute in a variety of pathophysiology in general, it is of great interest to determine which ROCK1 or ROCK2 inhibition could induce such hypotensive effects by Rip.

In the current study, to elucidate the role of ROCK1 and ROCK2 in terms of glaucomatous TM, the effects of pan-ROCK-i, Rip and the selective ROCK2 inhibitor (ROCK2-i), KD025 on several properties of the TGFβ2 treated 2D and 3D HTM cells the physical properties including, physical properties of the 3D sphenoid, size and stiffness of and the expression of major extracellular matrix (ECM), collagen (COL) 1, 4 and 6, fibronectin (FN) and α smooth muscle actin (αSMA), and their modulators, tissue inhibitors matrix proteinase (TIMP) 1–4, and matrix metalloproteinase (MMP) 2, 9 and 14 (2D and 3D) were investigated.

## Materials and methods

### Human trabecular meshwork (HTM) cells

Immortalized human trabecular meshwork (HTM) cells were purchased from Applied Biological Materials Inc., Richmond Canada. In advance to the current study described below, HTM cells used were confirmed to ensure truly TM cells by the up-regulation of the mRNA expression of myocilin in response to dexamethasone as according to the consensus recommendations for TM cells described by Keller et al^21^.

### Transendothelial electron resistance (TEER) measurements and the FITC-dextran permeability of 2D cultured HTM monolayer

TEER of the monolayered 2D cultured HTM cells were performed as described previously^22^ using a 12 well plate for TEER (0.4 μm pore size and 12 mm diameter; Corning Transwell, Sigma-Aldrich). Briefly, at approximately 80% confluence, 5 ng/mL TGFβ2 and ROCK-i, 10 μM Rip or KD025 were added the apical side of the wells (Day 1), and cultured until Day 6. At Day 6, the wells were washed twice with PBS, and TEER (Ωcm^2^) were measured using an electrode (KANTO CHEMICAL CO. INC., Tokyo, Japan)^20^.

In terms of fluorescein isothiocyanate (FITC)-dextran permeability, a 50 μmol/L solution of FITC-dextran (Sigma-Aldrich) was added to the well basal compartments of the culture and the culture medium from the apical compartment was collected at 60 min for the above different experimental conditions. The concentrations of the FITC-dextran were measured using a multimode plate reader (Enspire; Perkin Elmer, MA USA) at an excitation wavelength of 490 and an emission wavelength of 530 nm. The fluorescence intensity of the control medium was used as the background concentration.

### 2D and 3D cultures of human trabecular meshwork (HTM) Cells

2D and 3D cultures of HTM cells were prepared as described in a previous report^20^. Briefly, the 2D cultured HTM cells were further processed for the 3D sphenoid culture using a hanging droplet spheroid (3D) culture plate (# HDP1385, Sigma-Aldrich) during 6 days. For evaluation of drug efficacy of ROCK-is on TGFβ2 treated 3D HTM sphenoids, at Day 1, 5 ng/mL TGFβ2 and ROCK-i, Rip or KD025 at several concentrations (0, 1, 10 or 100 μM) were added, and half of the medium (14 μL) in each well was exchanged daily.

### Solidity measurement of 3D sphenoid

The physical stiffness analysis of the 3D HTM sphenoids was performed using a micro-squeezer (MicroSquisher, CellScale, Waterloo, ON, Canada) as previously reported^20,23^. Briefly, under several conditions as above, a single sphenoid at Day 6 was moved onto a 3-mm square microplate, and compression plate was placed on the top of the sphenoid. Then, the 3D sphenoid was compressed by down-ward movement of the compression plate to cause a 50% deformity of the 3D sphenoid during 20 s under monitoring by a microscopic camera. The force required to achieve a 50% strain was measured through the cantilever, and the data are expressed as force/displacement (μN/μm).

### Quantitative PCR

Total RNA extraction, reverse transcription and following real-time PCR with the Universal Taqman Master mix using a StepOnePlus instrument (Applied Biosystems/Thermo Fisher Scientific) were performed as describe previously^20^. The respective cDNA values are shown as fold-change relative to the control of normalized housekeeping gene 36B4 (*Rplp0*). Sequences of primers and Taqman probes used are as described in supplemental Table 1.

### Immunocytochemistry of 3D HTM sphenoids

Immunocytochemistry of the 3D HTM sphenoids was examined by previously described methods, with minor modifications^20^. Unless otherwise stated, all procedures were performed at room temperature. Briefly, 3D HTM sphenoids prepared as described above under several experimental conditions were fixed in 4% paraformaldehyde in phosphate buffered saline (PBS) overnight, blocked in 3% BSA in PBS for 3 h and then washed twice with PBS for 30 min. Thereafter, they were incubated with an anti-human COL1, COL4, COL6 or FN rabbit antibody (1:200 dilutions) and incubated at 4 °C overnight. After washing 3 times with PBS for 1 h each, they were then reacted with a 1:1000 diluted goat anti-rabbit IgG (488 nm), phalloidin (594 nm) and DAPI for 3 h, followed by mounting with ProLong Gold Antifade Mountant with a cover glass. Immunofluorescent images were obtained by Nikon A1 confocal microscopy using a × 20 air objective with a resolution of 1024 × 1024 pixels. For 3D sphenoids, serial-axis images with a 2.2 μm interval at a 35 μm height from their surface were obtained. The maximum intensity/surface area among above the observed areas was calculated using Image J (NIS-Elements 4.0 software) as follows: surface area = D × A/(A + π × H2), where D (μm) indicates sphenoid diameter, A (μm^2^) indicates area of sectioned sphenoid, and H (μm) indicates height (= 35 μm). For estimation of the numbers of cells present within a 3D sphenoid, the volume of a 3D sphenoid and the volume of a representative cell was calculated by assuming a spherical shape and the tentative diameters were estimated by largest cross-section of phalloidin images of the 3D sphenoid (n = 5) and the distance between two adjacent nuclei stained by DAPI (n = 5 for one section. These calculations were repeated five times using different preparations), respectively.

### Gelatin-zymography

Gelatin-zymography of the culture medium obtained from 3D HTM sphenoid culture as above was performed using gelatin-zymography kit (AK47, Cosmo Bio Co., Ltd. Tokyo Japan) according to the manufacturer’s protocol. After staining with Coomassie Brilliant Blue R-250 and de-staining, quantification of pro-MMP2 and pro-MMP9 bands were evaluated by quantified using Quantity One (version 4.5, BIO-RAD).

### Statistical analysis

By statistical analyses using Graph Pad Prism 8 (GraphPad Software, San Diego, CA), statistical significance with a confidence level greater than 95% by a two-tailed Student’s t-test or two-way analysis of variance (ANOVA) followed by a Tukey’s multiple comparison test was performed as described previously^20^.

## Results

**Figure 1:**
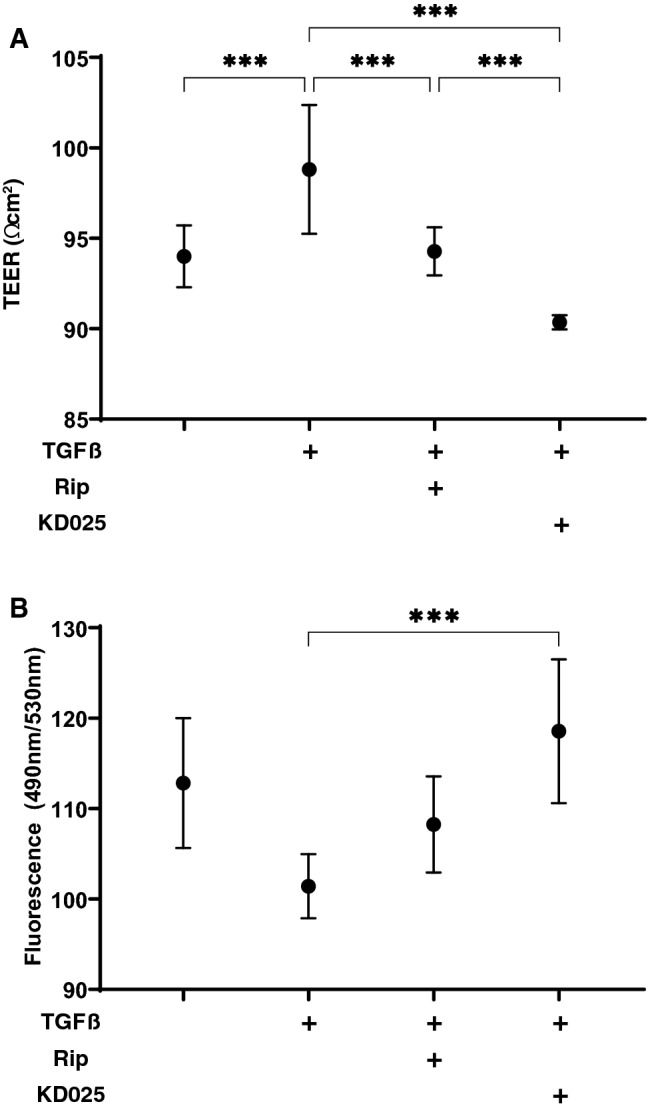
Effects of ROCK-is on transendothelial electrical resistance (TEER) (**A**) and FITC-dextran permeability (**B**) of TGFβ2 treated 2D culture of HTM cell monolayer. To evaluate the effects of 10 μM ripasudil (Rip) or KD025 on the barrier function (Ωcm^2^) and the permeability of TGFβ2 untreated or treated 2D cultured HTM monolayers, TEER (panel **A**) and FITC-dextran permeability (panel **B**) measurements were performed, respectively. All experiments were performed in triplicate using fresh preparations (n = 4). Data are presented as the arithmetic mean ± standard error of the mean (SEM). ***P < 0.005 (ANOVA followed by a Tukey’s multiple comparison test).

To compare pan ROCK-i, ripasudil (Rip) and ROCK2-i, KD025 toward TGFβ2 treated HTM cells, the barrier function by transendothelial electron resistance (TEER) measurements and the FITC-dextran permeability of 2D cultured HTM cell monolayers was evaluated. As shown in Fig. 1, the TEER values and FITC-dextran permeability were substantially increased and decreased, respectively, upon exposure to 5 ng/ml TGFβ2, and the TGFβ2 induced effects were significantly suppressed by ROCK-is, in which their suppressive efficacy was more evident in KD025 than Rip. Thus, this result indicates that such suppressive effects toward TGFβ2 induced an increase in the TEER value and a decrease of the FITC-dextran permeability in the 2D cultured HTM and that this inhibition is caused by ROCK2 rather than ROCK1.

**Figure 2:**
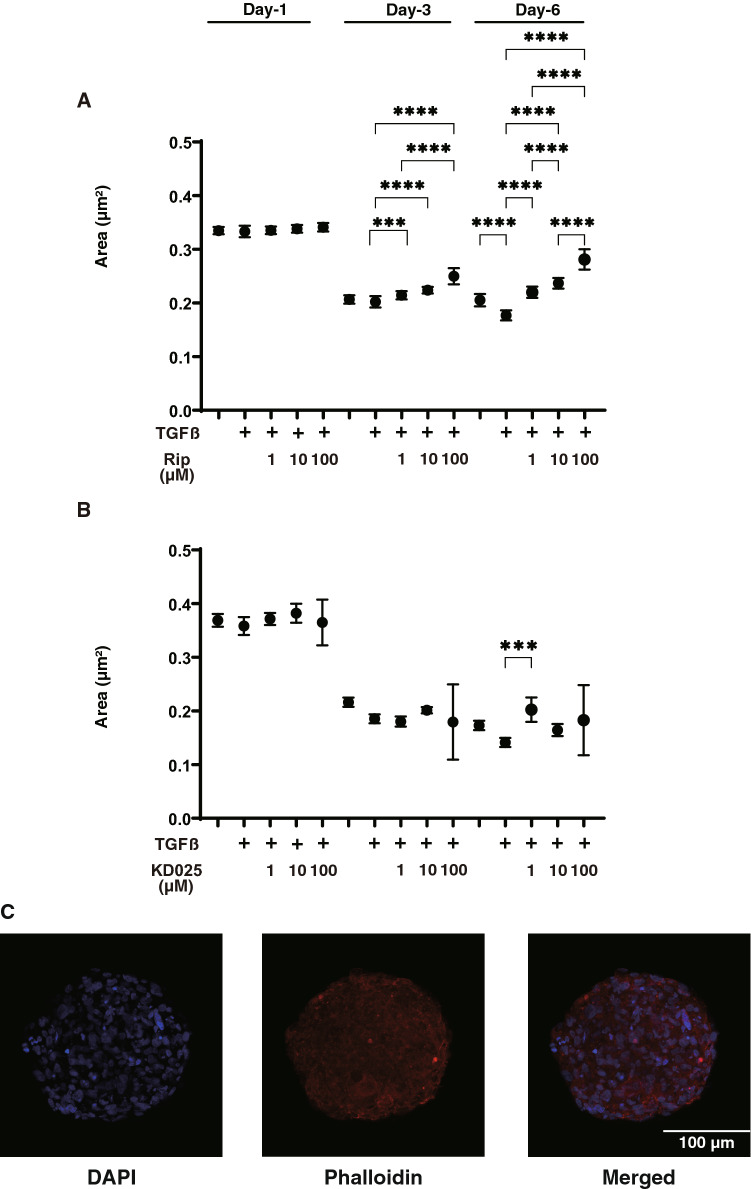
Changes in TGFβ2 treated 3D HTM sphenoid size at Day 1, 3 or 6 in the presence and absence of different concentrations of ripasudil (**A**) or KD025 (**B**), and representative non-treated 3D sphenoid image stained with DAPI and phalloidin (**C**). At Day 1, 3 or 6, the mean sizes of HTM 3D sphenoids (non-treated control) and those treated by 5 ng/ml TGFβ2 were plotted in the absence or presence of 1, 10 or 100 μM ripasudil (Rip, panel **A**) or KD025 (panel **B**). In panel (**C**), representative immunolabeling image of the non-treated 3D HTM sphenoid at Day 6 stained by DAPI and phalloidin. These experiments were performed in triplicate using fresh preparations (n = 10 and 5 for size measurement and immunolabeling, respectively). Data are presented as the arithmetic mean ± standard error of the mean (SEM). ***P < 0.005, ****P < 0.001 (ANOVA followed by a Tukey’s multiple comparison test).

**Figure 3:**
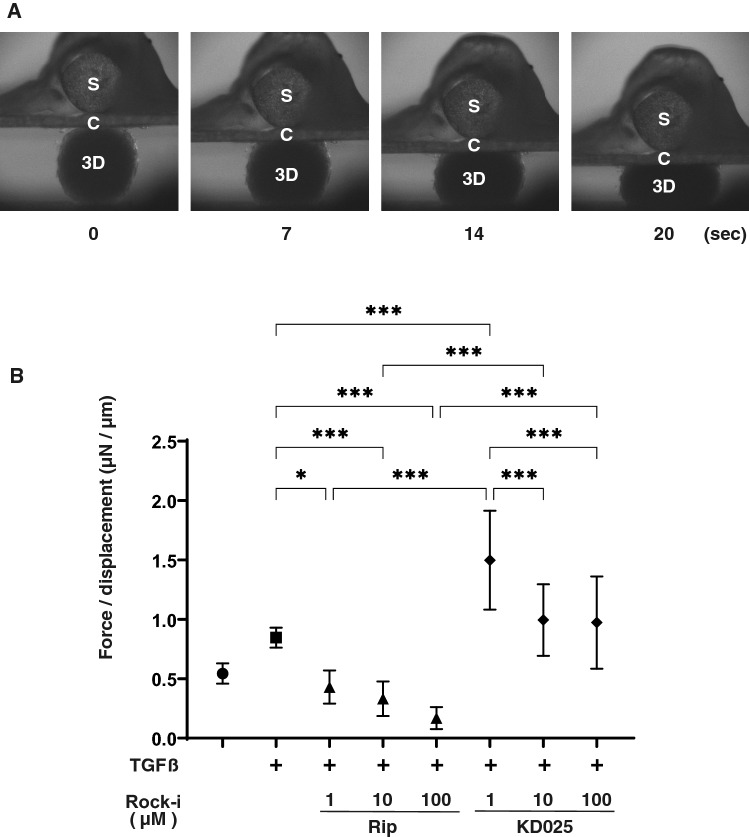
Physical solidity of 3D HTM sphenoids. The panel A shows representative photos demonstrating the micro-squeezer analysis at different time points; 0, 7, 14 or 20 (*S* pressure sensor, *C* compression plate, *3D* 3D sphenoid) measured over a period of 20 s. The force (μN) required to induce a 50% deformity of every single out of 15–20 freshly prepared 3D HTM sphenoids (non-treated control) and those treated by 5 ng/ml TGFβ2 in the absence or presence of 1, 10 or 100 μM ROCK-i, ripasudil (Rip) or KD025 were measured, and force/displacement (μN/μm) values were plotted in Panel B. *P < 0.05, ***P < 0.005 (ANOVA followed by a Tukey’s multiple comparison test).

To study this issue further, we evaluated the effects of pan-ROCK-i, Rip and ROCK2-i, KD025 on physical properties, size and stiffness, and mRNA expressions of ECM and their regulatory factors including TIMP and MMP of a recently established in vivo 3D HTM sphenoid culture model replicating glaucoma TM^20^ in addition to the conventional 2D culture of HTM cells. As shown in Figs. 2 and 3D HTM sphenoid sizes became substantially smaller during 6-day 3D HTM culture, and those were further enhanced by 5 ng/ml TGFβ2 at Day 6. In the presence of Rip, their 3D HTM sphenoid sizes became significantly larger in a concentration dependent manner at Day 3 and Day 6, although such enlarging effects were not observed in KD025 (Fig. 2A,B). To exclude the possibility that these down-sizing processes might be simply artifacts or cell death within the inside of the 3D HTM sphenoid, nuclear staining by DAPI was performed. As shown in the representative image of the non-treated control 3D HTM sphenoid (Fig. 2C), multiple layers of HTM cells that were arranged concentrically were observed within the 3D HTM sphenoid. Since our previous study demonstrated that intercellular interactions of the 3D sphenoids were much stronger than that for 2D cultured cells^24^, we were not able to count the numbers of cells present in the 3D sphenoid. Therefore, to estimate the numbers of cells in the 3D sphenoid, we calculated the volume of the overall 3D sphenoid and then calculated the numbers of cells within it, based on their volumes based on cross-section images. The results indicated that approximately, the cell numbers of each 3D sphenoid at Day 6 among three conditions were as follows: (non-treated control; 24,984.3 ± 4013, 5 ng/ml TGFβ; 26,104.2 ± 1995, 5 ng/ml TGFβ + 10 μM Rip; 24,610.4 ± 2592, 5 ng/ml TGFβ + 10 μM KD025; 20,834.9 ± 1591) were almost identical to those that were initially harvested (approximately 20,000 cells).

Concerning physical stiffness, upon the administration of 5 ng/ml TGFβ2, those of the 3D HTM sphenoids had increased significantly, and TGFβ2-induced increase of the stiffness were markedly suppressed by ROCK-is in a concentration dependent manner, whose efficacies were more evident in Rip than KD025 (Fig. 3). This result indicated ROCK1 inhibition by Rip caused significant enlargement and decreased stiffness of the TGFβ2-treated 3D HTM sphenoid, and ROCK2 inhibition by KD025 induced also their decrease stiffness but their efficacy was less than Rip.

**Figure 4:**
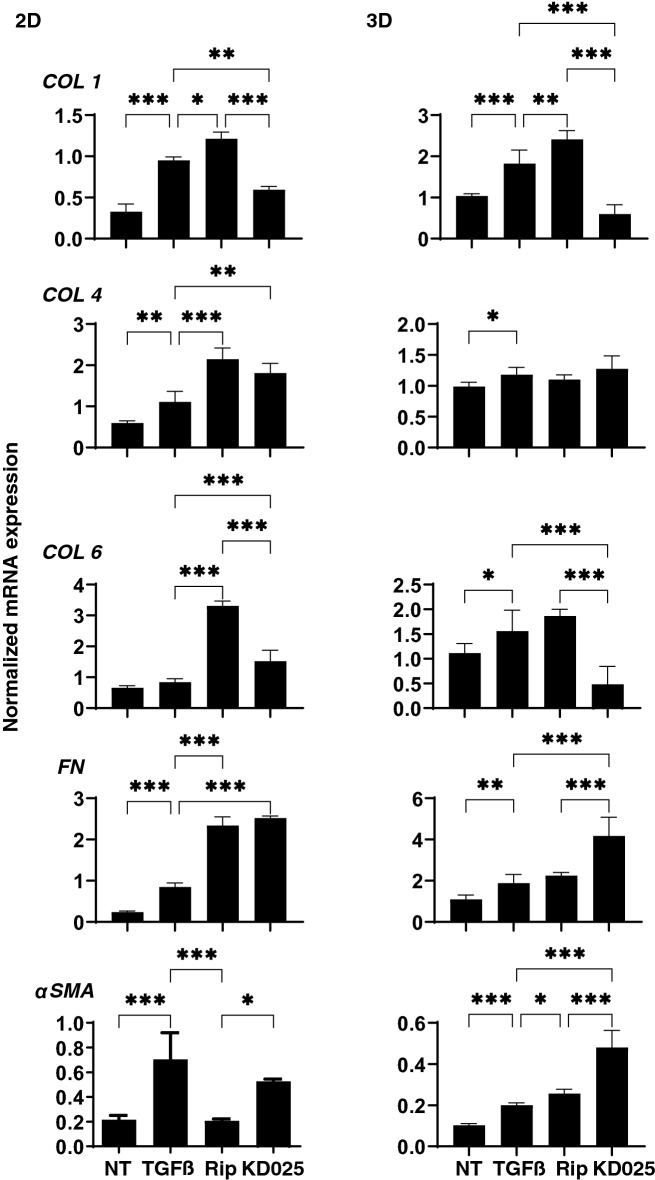
mRNA expression of ECM in 2D and 3D cultured HTM cells. At Day 6, HTM 2D cells and 3D sphenoids (control) and those treated by 5 ng/ml TGFβ2 in the absence or presence of 10 μM ripasudil (Rip) or KD025 were subjected to qPCR analysis to estimate the expression of mRNA in ECMs (*COL1*, *COL4*, *COL6*, *FN* and *aSMA*). All experiments were performed in duplicate using 15–20 freshly prepared 3D HTM sphenoids in each experimental condition. Data are presented as the arithmetic mean ± standard error of the mean (SEM). *P < 0.05, **P < 0.01, ***P < 0.005 (ANOVA followed by a Tukey’s multiple comparison test).

**Figure 5:**
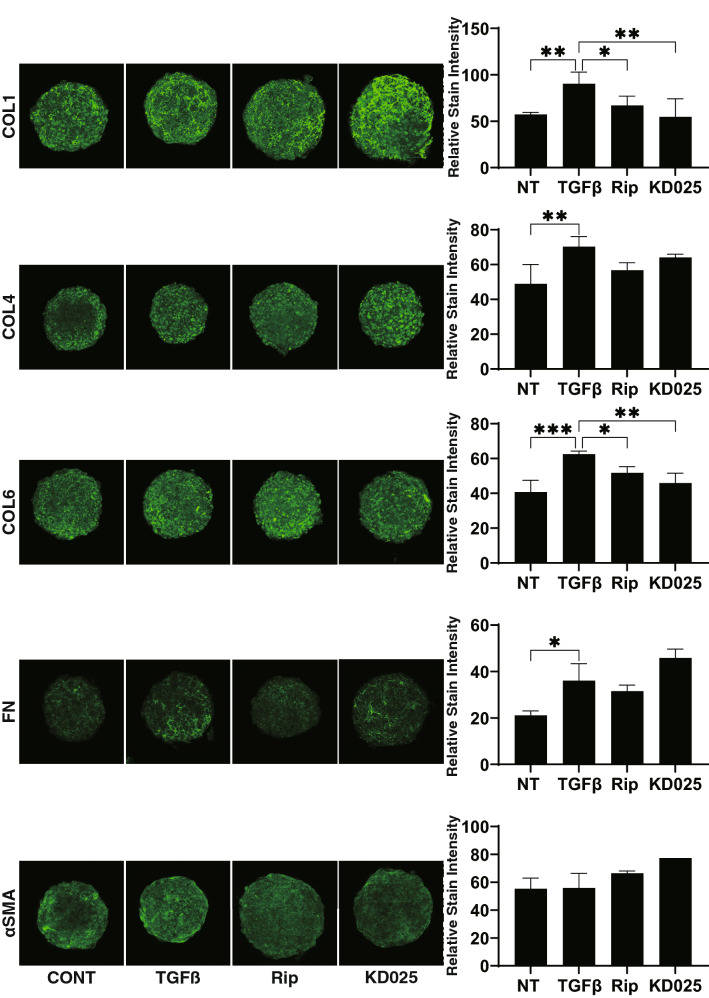
Immunostaining of ECMs of the 3D HTM sphenoids. At Day 6, 3D HTM sphenoid (non-treated control) and those treated by 5 ng/ml TGFβ2 in the absence or presence of 10 μM ripasudil (Rip) or KD025 were subjected to immunostaining for *COL 1*, *COL 4*, *COL 6*, *FN* and *a-SMA*. All experiments were performed in duplicate using fresh preparations (n = 4). Representative images are shown in left panels and relative staining intensities were plotted in right panels. Data are presented as the arithmetic mean ± standard error of the mean (SEM). * *P* < 0.05, ** *P* < 0.01, *** *P* < 0.005 (ANOVA followed by Tukey’s multiple comparison test).

To elucidate underlying mechanisms causing such ROCK-is effects as above, the mRNA expression of ECMs including *COL1, 4*, and *6, FN*, and *αSMA* were investigated (Fig. 4). Upon administering a 5 ng/ml solution of TGFβ2, expressions of all ECMs (3D) or all except *COL6* (2D) were significantly up-regulated. Addition of Rip induced further increase of *COL1* (2D and 3D), *COL4* (2D), *COL6* (2D), *FN* (2D) and *αSMA* (3D), or significant down-regulation of *αSMA* (2D). In contrast, the addition of KD025 induced substantially down-regulation of *COL1* (2D and 3D) and *COL6* (3D) and further up-regulation of *COL4* and *6* (2D), *FN* (2D and 3D) and *αSMA* (3D). Similarly, immunostaining of the 3D sphenoid indicated a significant increase in the staining intensities of all ECMs, except for αSMA, upon exposure to 5 ng/ml TGFβ2, and the effects of COL1 and COL6 were inhibited by ROCK-is (Fig. 5).

**Figure 6:**
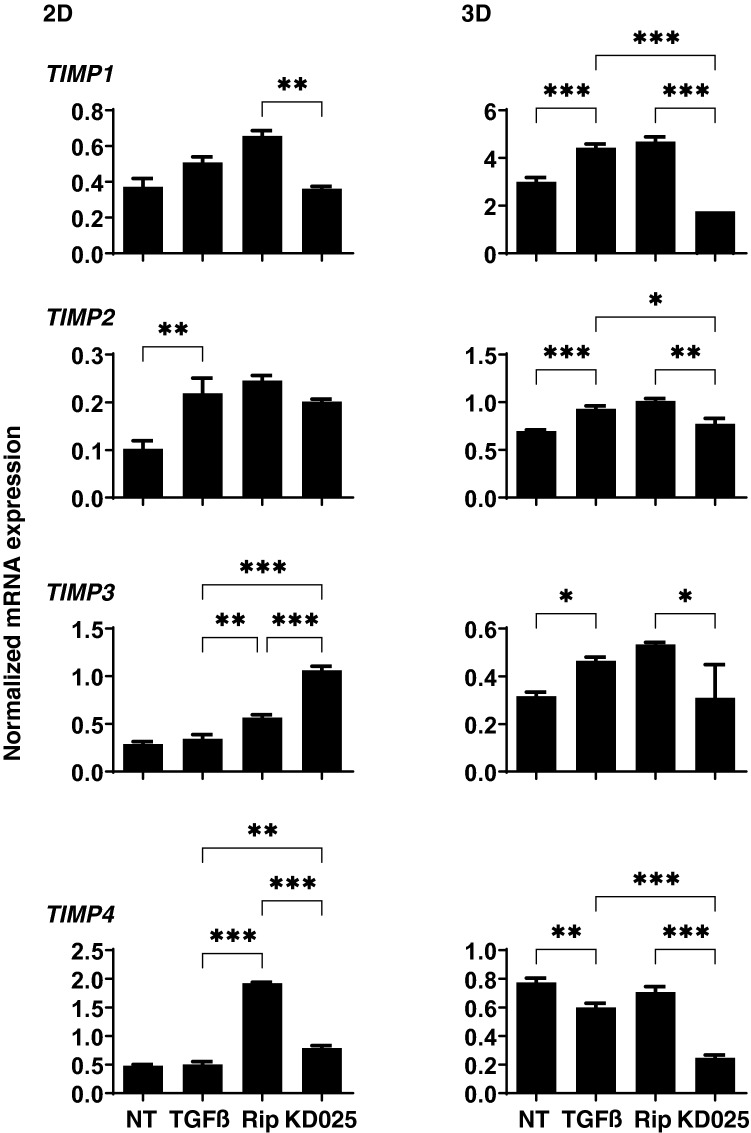
mRNA expression of TIMPs in 2D and 3D cultured HTM cells. At Day 6, HTM 2D cells and 3D sphenoids (control) and those treated by 5 ng/ml TGFβ2 in the absence or presence of 10 μM ripasudil (Rip) or KD025 were subjected to qPCR analysis to estimate the expression of mRNA in *TIMP1-4*. All experiments were performed in duplicate each using 15–20 freshly prepared 3D HTM sphenoids in each experimental condition. Data are presented as the arithmetic mean ± standard error of the mean (SEM). *P < 0.05, **P < 0.01, ***P < 0.005 (ANOVA followed by a Tukey’s multiple comparison test).

**Figure 7:**
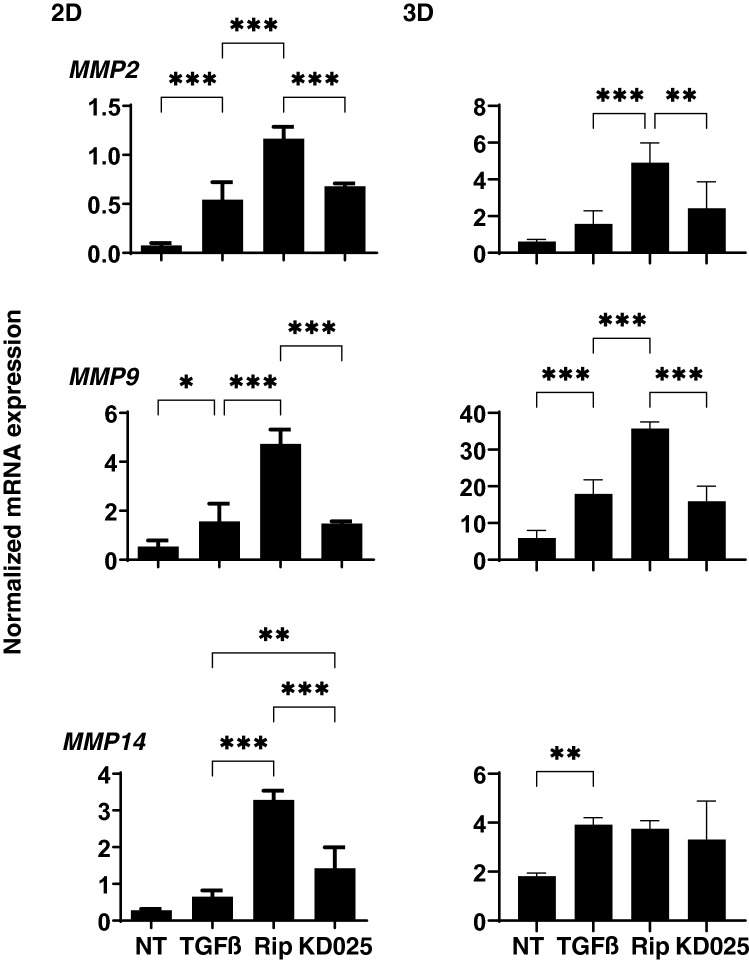
mRNA expression of MMPs in 2D and 3D cultured HTM cells. At Day 6, HTM 2D cells and 3D sphenoids (control) and those treated by 5 ng/ml TGFβ2 in the absence or presence of 10 μM ripasudil (Rip) or KD025 were subjected to qPCR analysis to estimate the expression of mRNA in *MMP 2, 9* and *14*. All experiments were performed in duplicate each using 15–20 freshly prepared 3D HTM sphenoids for each experimental condition. Data are presented as the arithmetic mean ± standard error of the mean (SEM). *P < 0.05, **P < 0.01, ***P < 0.005 (ANOVA followed by a Tukey’s multiple comparison test).

**Figure 8:**
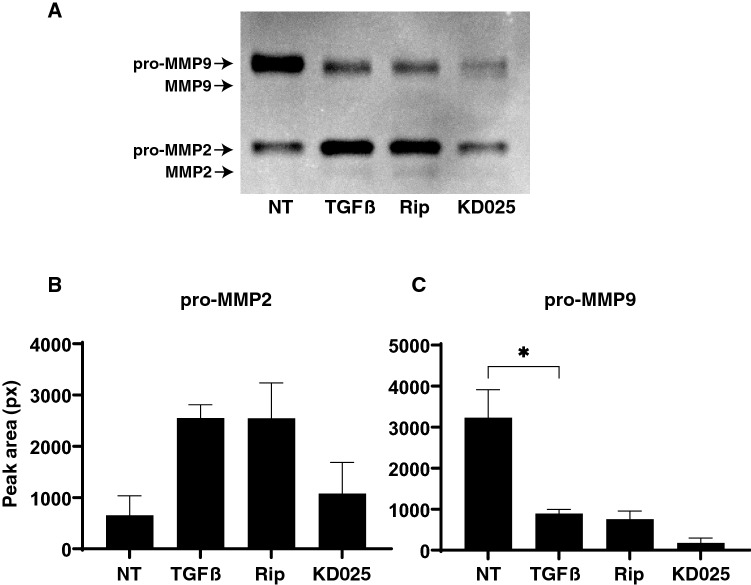
Gelatin-zymography of 3D HTM sphenoids. At Day 6, culture medium 7.5 out of 28 μl obtained from HTM 3D sphenoids and those treated by 5 ng/ml TGFβ2 in the presence of 10 μM ripasudil (Rip) or KD025 were subjected to gelatin-zymography. Representative their monochrome inversion image is shown in panel (**A**). The areas of the bands corresponding to pro-MMP2 (panel **B**) or pro-MMP9 (panel **C**) were qualified and plotted. All experiments were performed in duplicate using fresh preparations. Data are presented as the arithmetic mean ± standard error of the mean (SEM). *P < 0.05 (ANOVA followed by a Tukey’s multiple comparison test).

To study further, qPCR analysis of *TIMP1-4* and *MMP2, 9* and *14* (2D and 3D), and zymography (3D) were performed. As shown in Figs. 6 and 7, *TIMP1* (3D), *TIMP2* (2D and 3D) and *TIMP3* (3D), and *MMP2* (2D), *MMP9* (2D and 3D) and *MMP14* (3D) were significantly up-regulated, and *TIMP4* (3D) were markedly down-regulated upon administering a 5 ng/ml solution of TGFβ2. Addition of Rip or KD025 induced significant up-regulation of *MMP2* (2D and 3D), *MMP9* (2D and 3D) and *MMP14* (2D), or down-regulation of *TIMP1*, *2* and *4* (3D) and up-regulation of *TIMP3, TIMP4* and *MMP14* (2D), respectively. Different effects toward pro-MMP2 and pro-MMP9 between Rip and KD025 were also observed by gelatin-zymography (3D) (Fig. 8). Similar observation that pro MMPs were much more abundant as compared to the active forms in zymography in human TM was also reported in the previous study^25^. These observations indicated Rip and KD025 differently affected toward gene expressions of ECM and their modulators, MMPs and TIMPs, and those were also different between 2 and 3D culture of HTM.

## Discussion

Decreasing IOP to suitable levels by anti-glaucoma medication, laser treatment or surgery is well known to be the only evidence-based therapy for glaucomatous optic neuropathy (GON)^26–29^. In terms of maintenance of the IOP levels, the aqueous humor (AH) production and drainage through conventional roots by the TM and the uveoscleral root, in which approximately 70–90% and 10–30%, of the AH is drained, respectively are precisely regulated^30^. The excess deposition of extracellular matrix (ECM) molecules such as collagens (COLs), fibronectin (FN) and others to TM appears to cause elevation of IOPs in both primary open glaucoma (POAG) as well as steroid-induced glaucoma (SG)^31^ based upon several studies using animal model as well as TM cell culture^19,32^.

In contrast to the conventional 2D cell culture, the 3D sphenoid cell cultures has recently received more attention for application for suitable disease ex vivo diseases models including a steroid-induced glaucoma^33,34^. Vernazza et al. compared chronic stress exposure induced cellular responses between the 2D and 3D cultured HTM, and claimed that the 3D-cultured HTM cells are much better in the sensitivities against intracellular reactive oxidative species production induced by a hydrogen peroxide treatment^35^. Nevertheless, such 3D culture techniques may have some difficulties in terms of mimicking the physiological and pathological conditions of human TM due to the presence of unnecessary scaffolds. To avoid such unnecessary scaffolds, we recently developed a 3D cell drop culture method as ex vivo models for Graves’ orbitopathy^23^, deepening upper eyelid sulcus (DUES)^24,36^. In our earlier pilot study using this method, we found that TGFβ2 significantly induced the down-sizing and stiffness of 3D HTM sphenoids, and those effects were substantially inhibited by pan-ROCK-is^20^.

ROCK1 and ROCK2 were initially recognized as ubiquitously expressed proteins throughout embryogenesis through adult tissues^6,37^. Although both ROCK1 and ROCK2 appear to function in similar roles including focal adhesion formation and microfilament bundling, siRNA knockdown has revealed that there is some difference in their functions. In fibroblasts, ROCK1 and ROCK2 are differently involved in the assembly of fibronectin matrices at the cell surface during actin cytoskeleton mediated extracellular matrix assembly^38,39^. In addition, ROCK1 knockdown in keratinocytes decreased cell adhesion to fibronectin, although knockdown of the ROCK2 promoted fibronectin adhesion^40,41^. These observations indicate that ROCK1 and ROCK2 may have different roles and localization in various tissues and organs. In fact, only ROCK1 is cleaved by caspase-3 during apoptosis^42,43^ while smooth muscle-specific basic calponin is phosphorylated by ROCK2, but not ROCK1^44^. Expressed sequence tag (EST) analysis using the Tissue-specific Gene Expression and Regulation (TiGer) database^45^ demonstrate that ROCK1 and ROCK2 distribute similarly but, substantially different in some few specific organ and/or tissues. In fact, there was predominantly ROCK1 expression in the thymus and blood, with little to no ROCK2 expression, while in contrast, ROCK2 is most highly expressed within cardiac and brain tissues in addition to eye^6,46,47^. Thus, taking these facts into account, we rationally speculated that ROCK-is induced hypotensive effects toward IOP may be caused by their inhibition toward ROCK2 rather than ROCK1. However as of this writing, little information has been available. In the present study, to elucidate contribution of ROCK1 and ROCK2 inhibition within ROCK-is induced hypotensive efficacy, effects of pan-ROCK-i, Rip and specific inhibitor for ROCK2, KD025 were compared with each other using a recently developed TGFβ2 treated 3D HTM sphenoid culture as an ex vivo model for glaucomatous HTM and obtained following observations; (1) suppressive effects toward TEER of the 2D HTM monolayer; KD025 > Rip, (2) enlargement of the 3D HTM sphenoid; Rip > KD025, and (3) decrease in the stiffness of the 3D HTM sphenoid; Rip > KD025. Therefore, based upon our present observations, we rationally speculated that ROCK1 and ROCK2 may be related to physical properties of 3D structure and permeability of HTM, respectively. Furthermore, this our speculation may also be supported by the present data that Rip and KD025 differently altered expressions of ECM and their modulators, TIMPs and MMPs.

ECM is recognized as not only a structural support within organs but also a specific modulator for cell–cell signals as well as a regulator in a variety of cellular functions^48^. Among the ECM, COL1, and COL4 and COL6 are major components of the basement membrane (BM)^49–52^, and FN is an important molecule defining cell shape and contractility in association with COL1^53^. In terms of the fibrotic changes of TM cells, TGFβ2 activates cytoplasmic Smad2/3^54,55^ leading up-regulation of ECM expressions, such as FN and COL4 which induced impediment to AH outflow through the TM resulting in elevation of the IOP levels^56^. MMPs are well established as having a major role in degrading ECM components during normal tissue remodeling and wound healing^57^. In a study using perfused human anterior segment organ cultures, it was demonstrated that the ease of AH outflow was increased or decreased by the addition of recombinant MMP2, MMP3, or MMP9 or the inhibition of endogenous MMP activity, respectively^58–60^. Furthermore, based upon an altered structural organization of the TM and the occurrence of early-onset ocular hypertension in MMP9 knockout mice, De Groef et al. suggested that the MMP9 dependent remodeling of the TM is required to enhance outflow and maintain IOP homeostasis^61^. Alternatively, the aqueous levels of MMP2 were significantly decreased in patients with POAG as compared to cataractous patients, while the aqueous levels of TIMP2 were unchanged^62^. In contrast, another similar study reported that the AH concentrations of MMP2 and TIMP2, TIMP2 levels were significantly increased in patients with POAG as compared with cataractous patients^63^. Thus, these collective results suggest that an imbalance between the MMP/TIMP ratio may contribute to the pathogenesis associated with the decreased outflow facility and elevated IOP in POAG^62,63^.

There were following several study limitations required to be discussed; (1) we performed current whole study using commercially available immortalized HTM cells. Although the provider certified that these are truly HTM cells in addition to test DEX-induced up-regulation in myocilin, it is revealed that there are significant biological variabilities from donor to donor, and thus additional study to characterize these cells to be TM in nature. Nevertheless, no permission has been available to use human donor eyes for research under our national laws. Therefore, an additional study using confirmed glaucomatous HTM cells without any stimulus of TGFβ2 from several different human donors may be needed. (2) In terms of the structural similarity between our 3D HTM sphenoids and in vivo human TM multiple-sheets structures, we found that the HTM cells are lined up concentrically and that multiple layers are then formed as shown in Fig. 2C, although this may be somewhat different from the “multiple sheet layers” found in human TM structures. Therefore, further investigations using confirmed glaucomatous TM cells and in vivo the use of anterior segment and mouse models will be required.

In conclusion, our present study not only facilitate a better understanding of the roles of ROCK1 and ROCK2 in the AH dynamics of HTM and molecular pharmacology of ROCK-is toward HTM, but also may suggests additional insights into potential future therapeutic strategies for the treatment of glaucoma.

## Supplementary Information

Supplementary Information.
