# Supplementary material for: Diverse effects of pan-ROCK and ROCK2 inhibitors on 2 D and 3D cultured human trabecular meshwork (HTM) cells treated with TGFβ2
Source: Sci Rep. 2021 Jul 27;11:15286. doi: 10.1038/s41598-021-94791-4 (PMC8316578; doi:10.1038/s41598-021-94791-4)
Supplement: Supplementary file 1 — Supplementary Information. [file 41598_2021_94791_MOESM1_ESM.pdf]

		Sequence	Exon Location	RefSeqNumber
human RPLP0	Probe	5’-/56-FAM/CCCTGTCTT/ZEN/CCCTGGGCATCAC/3IABkFQ/-3’	2-3	NM_001002
	Primer2	5’-TCGTCTTTAAACCCTGCGTG-3’		
	Primer1	5’-TGTCTGCTCCCACAATGAAAC-3’		
human COL1A1	Probe	5’-/56-FAM/TCGAGGGCC/ZEN/AAGACGAAGACATC/3IABkFQ/-3’	1-2	NM_000088
	Primer2	5’-GACATGTTCAGCTTTGTGGAC-3’		
	Primer1	5’-TTCTGTACGCAGGTGATTGG-3’		
human COL4A1	Probe	5’-/56-FAM/TCATACAGA/ZEN/CTTGGCAGCGGCT/3IABkFQ/-3’	51-52	NM_001845
	Primer2	5’-AGAGAGGAGCGAGATGTTCA-3’		
	Primer1	5’-TGAGTCAGGCTTCATTATGTTCT-3’		
human COL6A1	Primer2	5’-CCTCGTGGACAAAGTCAAGT-3’	2-3	NM_001848
	Primer1	5’-GTGAGGCCTTGGATGATCTC-3’		
human FN1	Primer2	5’-CGTCCTAAAGACTCCATGATCTG-3’	3-4	NM_212482
	Primer1	5’-ACCAATCTTGTAGGACTGACC-3’		
human αSMA	Probe	5’-/56-FAM/AGACCCTGT/ZEN/TCCAGCCATCCTTC/3IABkFQ/-3’	8-9	NM_001613
	Primer2	5’-AGAGTTACGAGTTGCCTGATG-3’		
	Primer1	5’-CTGTTGTAGGTGGTTTCATGGA-3’		
human TIMP1	Probe	5’-/56-FAM/TCAACCAGA/ZEN/CCACCTTATACCAGCG/3IABkFQ/-3’	2-4	NM_003254
	Primer2	5’-CCTTCTGCAATTCCGACCT-3’		
	Primer1	5’-GCTTGGAACCCTTTATACATCTTG-3’		
human TIMP2	Probe	5’-/56-FAM/TCTCATTGC/ZEN/AGGAAAGGCCGAGG/3IABkFQ/-3’	3-4	NM_003255
	Primer2	5’-GACGTTGGAGGAAAGAAGGA-3’		
	Primer1	5’-TGTGGTTCAGGCTCTTCTTC-3’		
human TIMP3	Probe	5’-/56-FAM/CCTCCTTTA/ZEN/CCAGCTTCTTCCCCAC/3IABkFQ/-3’	1-3	NM_000362
	Primer2	5’-CCTTCTGCAACTCCGACATC-3’		
	Primer1	5’-CGGTACATCTTCATCTGCTTGA-3’		
human TIMP4	Probe	5’-/56-FAM/ACTGAGGAC/ZEN/CTGACCAGTCAAGAGA/3IABkFQ/-3’	3-4	NM_003256
	Primer2	5’-GGTTTGAGAAAGTCAAGGATGTTC-3’		
	Primer1	5’-GTTGCACAGATGGATGAAGAC-3’		
human MMP2	Primer2	5’-TCCACCACCTACAACTTTGAG-3’	6-7	NM_004530
	Primer1	5’-GTGCAGCTGTCATAGGATGT-3’		
human MMP9	Primer2	5’-ACATCGTCATCCAGTTTGGTG-3’	3-4	NM_004994
	Primer1	5’-CGTCGAAATGGGCGTCT-3’		
human MMP14	Primer2	5’-TTCGCCGACTAAGCAGAAG-3’	1-1	NM_004995
	Primer1	5’-CTTGAATTCCTAGACCGCTGT-3’		

Supplemental Table 1 Sequences of primers of qPCR

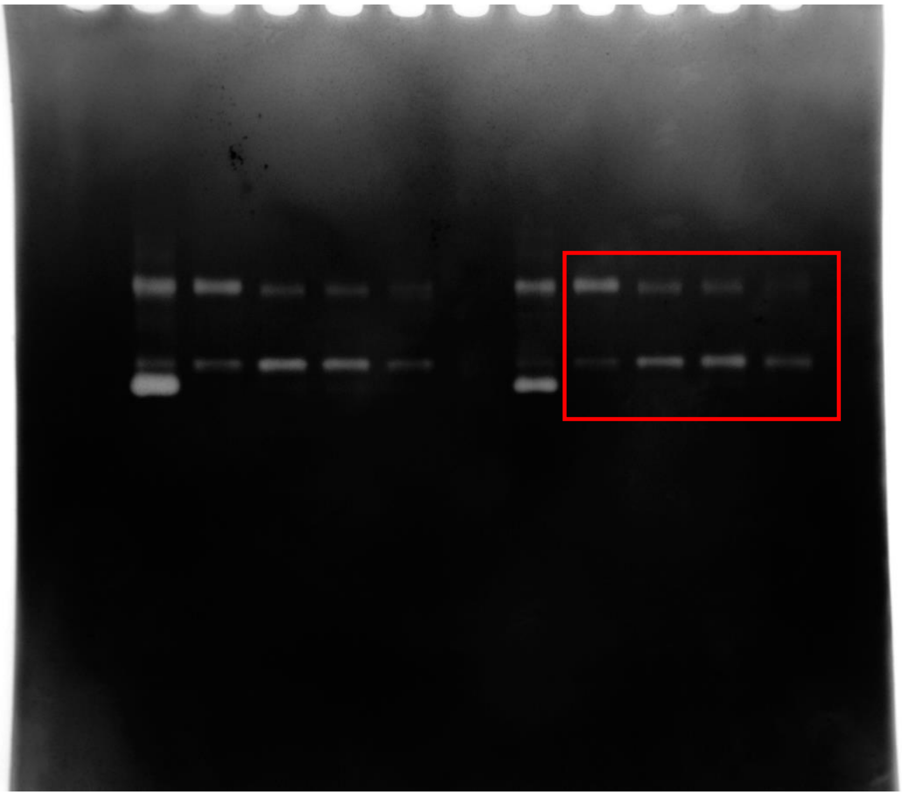

Supplemental information for zymography

Fig. 8 is the monochrome inversion image obtained from original zymography gel attached here (red box).
